# Systematic Review of Diagnostic Sensors for Intra-Abdominal Pressure Monitoring

**DOI:** 10.3390/s21144824

**Published:** 2021-07-15

**Authors:** Chien-Hung Liao, Chi-Tung Cheng, Chih-Chi Chen, Yu-Hsin Wang, Hsin-Tzu Chiu, Cheng-Chun Peng, Uei-Ming Jow, Yen-Liang Lai, Ya-Chuan Chen, Dong-Ru Ho

**Affiliations:** 1 Department of Trauma and Emergency Surgery, Linkou Chang Gung Memorial Hospital, Chang Gung University, Taipei 10547, Taiwan; surgymet@gmail.com (C.-H.L.); atong89130@gmail.com (C.-T.C.); D01548012@ntu.edu.tw (Y.-H.W.); jessie10723@gmail.com (H.-T.C.); lopengve0730@gmail.com (C.-C.P.); jow0209@gmail.com (U.-M.J.); snoobe@gmail.com (Y.-L.L.); joycechen108@gmail.com (Y.-C.C.); 2 Department of Rehabilitation and Physical Medicine, Linkou Chang Gung Memorial Hospital, Chang Gung University, Taoyuan 33328, Taiwan; claudia5477@gmail.com; 3 Department of Urology, Chiayi Chang Gung Memorial Hospital, Chang Gung University, Chiayi 613016, Taiwan

**Keywords:** intraabdominal pressure, sensors, device, intraabdominal hypertension

## Abstract

Intra-abdominal pressure (IAP) is defined as the steady-state pressure within the abdominal cavity. Elevated IAP has been implicated in many medical complications. This article reviews the current state-of-the-art in innovative sensors for the measurement of IAP. A systematic review was conducted on studies on the development and application of IAP sensors. Publications from 2010 to 2021 were identified by performing structured searches in databases, review articles, and major textbooks. Sixteen studies were eligible for the final systematic review. Of the 16 articles that describe the measurement of IAP, there were 5 in vitro studies (31.3%), 7 in vivo studies (43.7%), and 4 human trials (25.0%). In addition, with the advancement of wireless communication technology, an increasing number of wireless sensing systems have been developed. Among the studies in this review, five presented wireless sensing systems (31.3%) to monitor IAP. In this systematic review, we present recent developments in different types of intra-abdominal pressure sensors and discuss their inherent advantages due to their small size, remote monitoring, and multiplexing.

## 1. Introduction

### 1.1. Introduction of Intra-Abdominal Pressure

Intra-abdominal pressure (IAP) is defined as the steady-state pressure within the abdominal cavity resulting from the interaction between the abdominal wall and viscera [1]. Elevated IAP has been implicated in many medical complications, including hernias, severe drops in cardiac output, and postoperative complications such as prolonged healing time and wound dehiscence. Furthermore, intra-abdominal pressure may be one of the few modifiable risk factors associated with developing a pelvic floor disorder [2]. Since the peritoneal cavity is a closed space, changes to its volume due to body position, muscle contraction, or respiration can change IAP, which is a key physiological process that occurs regularly during various activities [3]. Changes in IAP can cause a range of physiological and pathophysiological reactions, and elevated IAP in women is thought to be a risk factor for pelvic floor diseases, such as prolapse and incontinence [4]. One in eight women in the US will have surgery for pelvic organ prolapse and are advised to restrict physical activity to avoid elevating IAP [5].

Furthermore, persistent IAH can cause organ dysfunction in patients who have been treated in intensive care, surgery, trauma, and cardiology departments [6]. Elevated IAP decreases capillary blood flow in the viscera and can lead to a dismal prognosis in critical patients [7]. It is also increasingly recognized in patients after elective surgical procedures, including massive fluid resuscitation in cases of extra-abdominal trauma, severe burns, and major operations [8,9]. IAH has recently emerged as an issue of concern among critical care physicians [6,10,11]. Epidemiological studies have reported a highly variable incidence of IAH in critically ill patients, with values ranging from 31 to 59% [12,13,14]. Since the early 2000s, abdominal compartment syndrome (ACS), defined as an IAP above 20 mmHg, has been accepted as a well-defined clinical entity and occurs in conjunction with associated organ dysfunction [13,15]. In 2006, the World Society of Abdominal Compartment Syndrome (WSACS) defined IAH as IAP elevation above 12 mmHg in three consecutive measurements [16]. IAH is defined as a sustained or repeated pathological elevation of IAP > 12 mmHg. There are four grades of IAH, as described in Table 1.

It should be noted that the IAP ranges associated with these grades have been revised downward in recent years as the detrimental impact of elevated IAP on end-organ function has been recognized. Blaser et al. [11] reported that the presence and severity of IAH in the first 2 weeks of the ICU stay significantly and independently increased 28- and 90-day mortality. The overall effect of IAH on outcome may be a function of IAH severity because a higher IAH grade is associated with higher mortality and vice versa. The mortality can be as high as 38.6%. Therefore, routine monitoring of IAP is mandatory, particularly in critically ill patients in intensive care units. With an understanding of the risk factors for an increase in IAP and its progression to ACS, IAP monitoring has made it possible to detect early signs of IAH in patients under treatment [17]. Furthermore, early recognition of ACS from its clinical signs and risk factors can significantly reduce the associated morbidity and mortality. If clinical signs can be detected in time, emergent laparotomy can be performed immediately to relieve the pressure [18]. Since Kron et al. [19] first described a method in which the bladder wall is considered to act as a membrane pressure transducer, intravesical measurement with fluid-filled catheters has been well adapted to measure IAP. However, high variation in intravesical pressure measurements with patients in the required supine position makes it difficult for clinical physicians to monitor this parameter [20,21]. The fundamental purpose of a sensor system is to accurately measure a signal that reveals information about a patient’s well-being. The requirements for a particular pressure sensor technology depend strongly on (1) the area of interest, (2) the site of measurement, and (3) the method for which the sensor is employed [22]. Furthermore, fulfilling regulatory requirements for medical devices and quality systems is another consideration when developing new sensors in clinical practice [23,24]. Therefore, increasingly novel sensor designs have been developed to detect cavity pressure in the human body [22,25,26], and numerous new sensors to measure IAP have become available. Furthermore, with the advantage of wireless technology, new devices are frequently developed with wireless sensors. With these new tools, sensors can enable continuous monitoring and accurate measurement of IAP.

### 1.2. Principles of Pressure Sensors

**Figure 1:**
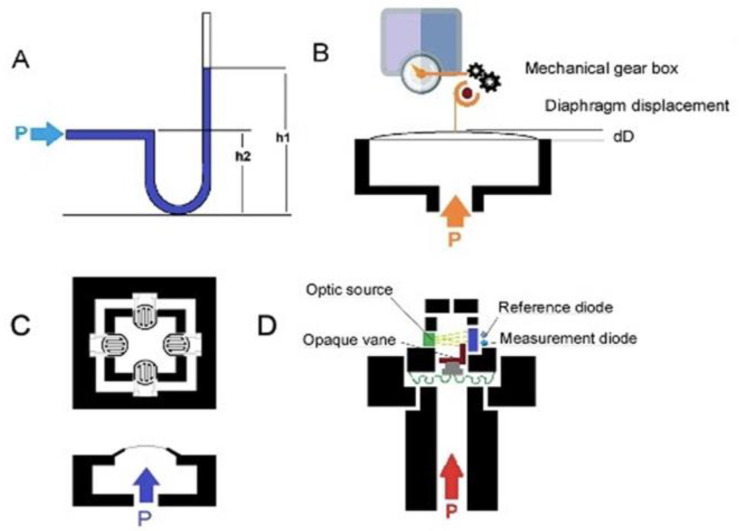
(**A**) Schematic of a water-/air-filled catheter as the pressure transducer; (**B**) schematic of a capacitive diaphragm sensor; (**C**) schematic of a piezoresistive diaphragm sensor; (**D**) schematic of optical fiber pressure sensors.

Given the increasing development of sensor systems, we performed a systematic review of all available studies published in English to thoroughly evaluate novel sensors that measure intra-abdominal pressure and their applications. Numerous types of sensors have been designed to measure the pressure of the intra-abdominal cavity. Four principles of pressure measurement are exemplified by the fluid-filled catheter sensor, classical strain gauge transducer, diaphragm displacement sensor, and optic-based sensors, as shown in Figure 1 [22].

Catheter-based pressure systems use a catheter filled with an incompressible medium (e.g., water or air) connected to a pressure sensor (Figure 1A). A change in pressure at the tip of the catheter compresses the fluid/air and transmits the pressure directly to the connected sensor [27]. IAP is measured indirectly via the patient’s intravesical pressure (IVP), which is obtained via the catheter. A change in pressure at the tip of the catheter compresses the fluid/air and transmits the pressure directly to the connected sensor [17]. In order to determine IVP from the catheter, the user must place the 0 mmHg mark of the manometer tube at the midaxillary line at the level of the iliac crest (future reference) and elevate the filter vertically above the patient. The user reads the IVP to determine the IAP when the meniscus has stabilized for at least 10 s. IAP is measured every 4 h or more frequently if it is greater than 12 mmHg or if the patient is hypotensive, has decreased urine output, or has a tense abdomen. IVP closely approximates IAP. Instillation of 50 mL of liquid into the bladder improves the accuracy of the IVP in measuring elevated IAPs [19]. The mean IAP difference for an individual is around 4 mmHg [28]. However, a clinically significant variation in this method is associated with side effects such as bowel perforation and peritonitis. It has been shown that IAP can be influenced by body position, with an increase in bladder pressure of up to 7.5 mmHg with a 45° positioning angle [28,29].

Diaphragm displacement sensors [27,30,31] (Figure 1B) have a bendable flat surface (i.e., the diaphragm) and are exposed to the pressure medium on one side. For a pressure sensor, the opposite side is a sealed cavity. When pressure is applied through the medium, the diaphragm deflects to an extent proportional to the magnitude of the pressure. Diaphragm displacement sensors provide isolation from process fluid and are good for low pressure. This sensor can be used to produce an electrically measurable response, such as resistance or capacitance changes proportional to the pressure. However, it is more expensive than other sensors.

In a capacitive diaphragm sensor, the diaphragm represents one electrode of a capacitor that has a fixed plate as the second electrode. Pressure-related deflection of the diaphragm reduces the separation of the electrodes, causing a capacitance change proportional to the applied pressure [30]. The electronic system can measure capacitance changes such as the resonant frequency to map the pressure.

In a piezoresistive diaphragm sensor, the transducers are made from a conductive material, and the strain gauge can be attached to a diaphragm that recognizes a change in resistance when the sensor element is deformed. Deformation causes a change in the output electrical resistance (Figure 1C). The resistance change can be most effectively measured using a Wheatstone bridge [32], which converts the variation to an output electrical signal. Silicon-based piezoresistive sensors used for IAP systems offer a good compromise between sensitivity and thermal characteristics. Moreover, silicon sensors, especially microelectromechanical system (MEMS) pressure sensors, can be integrated with an integrated circuit in a small package, and the sensor output, 10 mV/V, can be digitized. Although piezoresistive pressure sensors have the disadvantage of higher power consumption than some other types of pressure sensors, they have the advantage of robustness and high accuracy [33].

The optical fiber pressure sensor (Figure 1D) measures the variation in the received optical intensity due to source output power drifts, fiber movements, or the degradation of components in the system that contribute to the error in the measured pressure signal [34,35]. In an intensity-based optical pressure sensor, an increase in pressure causes the progressive blocking of the light source, and the sensor then measures the change in light received. Fiber-optic pressure sensors can be classified as either extrinsic, where the sensing takes place outside the fiber, or intrinsic, where the fiber itself changes in response to pressure. Very sensitive optical measurements can be made by exploiting interferometry: measuring the change in phase between light that has taken two different paths. This approach can detect changes in distance corresponding to a fraction of the wavelength of light. The optical sensors are not very sensitive to temperature changes, and hysteresis and repeatability errors are very low. Moreover, because of the small size and flexibility of fiber-optic sensors, they can be deployed in locations that would be hard to access with other techniques. Although the optical sensing element itself is passive, the light source detected by the sensors can be a problem, so most optical sensors are based on a wired system. In IBP and IAP applications [22,36], the reported optical system allows dual-channel detection, with probes having a typical sensitivity of 1.3 nm/kPa and an accuracy of 0.6 cm H_2_O. Table 2 compares the different sensor types.

Given the increasing development of sensor systems, we performed a systematic review of available studies published in English to thoroughly evaluate novel sensors that measure intra-abdominal pressure and their applications.

## 2. Materials and Methods

### 2.1. Literature Search

The systematic literature search was performed based on the preferred reporting items for systematic reviews and meta-analyses (PRISMA) statement [42]. A study was considered eligible if it evaluated a sensing measurement system or associated design, method, or materials for IAP monitoring for particular patient groups.

#### 2.1.1. Information Sources and Search

We attempted to identify all published studies that reported IAP-sensing systems. We searched the MEDLINE, EMBASE, and Google Scholar electronic databases. The search strategy restricted the language to English and the publication dates to between January 2010 and December 2019. The MEDLINE, EMBASE, and Google Scholar databases were searched using the following subject headings: “intra-abdominal pressure”, “intra-abdominal hypertension”, “abdominal compartment syndrome”, “sensor”, “measurement”, “device”,” intraperitoneal pressure”, and “abdominal trauma”. The bibliographies of relevant articles were also reviewed to identify additional studies. Inclusion criteria included original investigative studies and studies focusing on novel sensor development for IAP monitoring. Only articles in English were included. Because of the advance in technology, the diagnostic performance of medical devices and sensors has improved over the past decade. Therefore, we only searched for literature published after 2010. Authors whose names appeared in multiple studies that were otherwise eligible for inclusion were contacted to avoid any data duplication. We supplemented our search by manually reviewing the reference lists of all retrieved articles to identify other potentially relevant citations.

#### 2.1.2. Study Selection

Two independent reviewers (YL Lai and CC Chen) independently screened the titles, abstracts, and, if there was insufficient information in the abstract, full-text publications to determine the suitability of the studies for inclusion in the analysis. Studies evaluating the performance of IAP monitoring and sensor development were eligible if they provided detailed characteristics. Case reports, editorials, abstracts, and conference proceedings were excluded. In a second retrieval phase, references in original papers were examined for other publications according to the above terms, and these related articles were assessed. This procedure was repeated in two additional phases. An extensive manual search was also performed among publications and textbooks on diagnostic sensors and devices. Concept reviews and product brochures were excluded.

### 2.2. Data Collection and Validity Assessment

YL Lai and CC Chen independently extracted the study and sensor characteristics and the diagnostic accuracy of the IAP monitoring system. Cohen’s kappa coefficient (κ) was calculated to assess the agreement between review authors. No attempts to mask the authorship, journal name, or institution were made in this or any other step of the review process. Extracted data were entered into a database that was independently pre-prepared by both reviewers and compared at the end for consistency. Data extraction was performed by using spreadsheet software (Excel; Microsoft, Redmond, Wash). Any differences in opinion regarding inclusion were discussed with a third reviewer (CH Liao). Information about sensor model, study type, measurement route, monitor accuracy, the presence of wireless design, sensor stage, and target patient groups was collected. Finally, data were extracted from the studies using a data extraction sheet. The Quality Assessment of Diagnostic Accuracy Studies 2 (QUADAS-2) [43] checklist was used by two reviewers to assess the quality of the included studies. The statistical analyses were performed with Review Manager software, version 5.3 (the Nordic Cochrane Centre, the Cochrane Collaboration, Copenhagen, Denmark, 2014).

## 3. Results

### 3.1. Search Strategy

**Figure 2:**
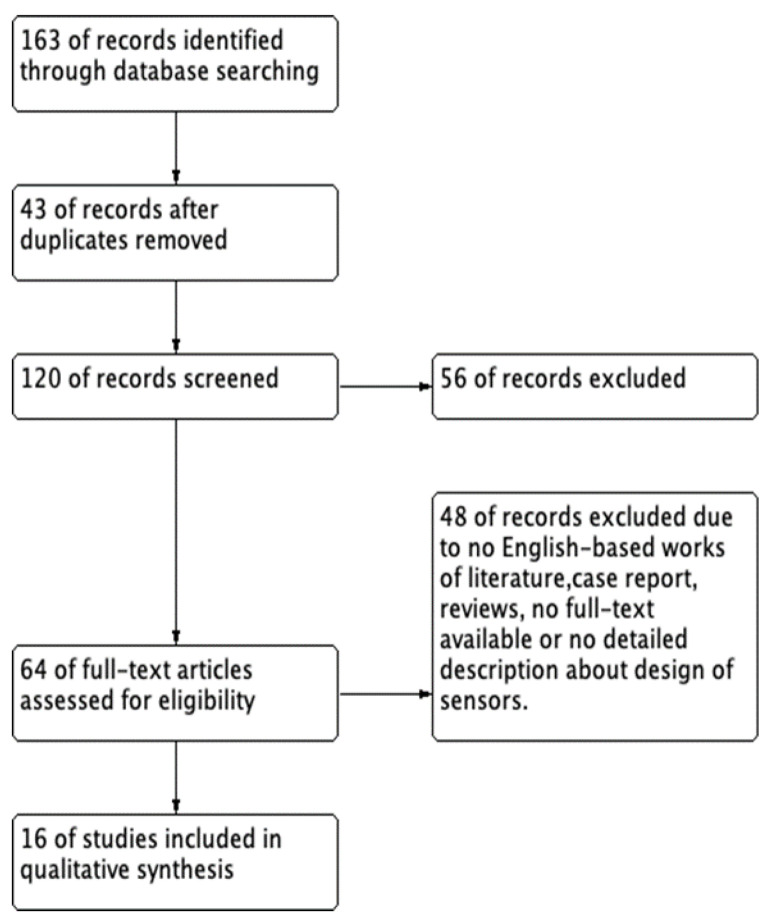
The flow diagram of this systematic review.

Using the search terms in MEDLINE, EMBASE, and Google Scholar electronic databases yielded a total of 163 potentially relevant studies. The flow diagram of this systematic review is presented in Figure 2.

We excluded 43 duplicate studies and 120 studies after applying the inclusion and exclusion criteria during the title and abstract screening. Fifty-six articles were excluded because of a lack of detailed IAP sensing data, and 64 articles were included in the full-text review. After reviewing the abstracts, 48 articles were excluded because they were not in English, were reviews, or the full text was not available. Finally, 16 articles [14,15,16,17,18,19,20,21,22,23,24,25,26,27,28,29] were included in the review of the characteristics. The 16 included studies are summarized in Table 3.

**Figure 3:**
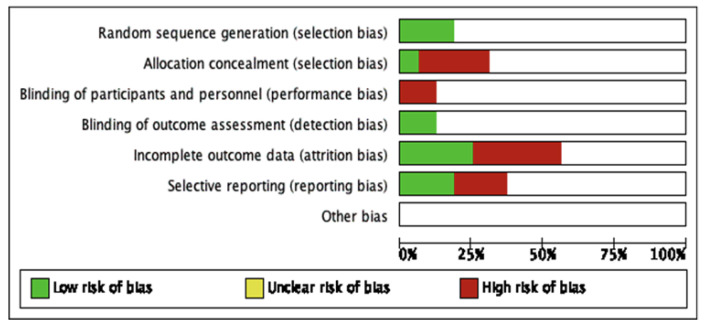
The summary of risk of bias of included studies.

**Figure 4:**
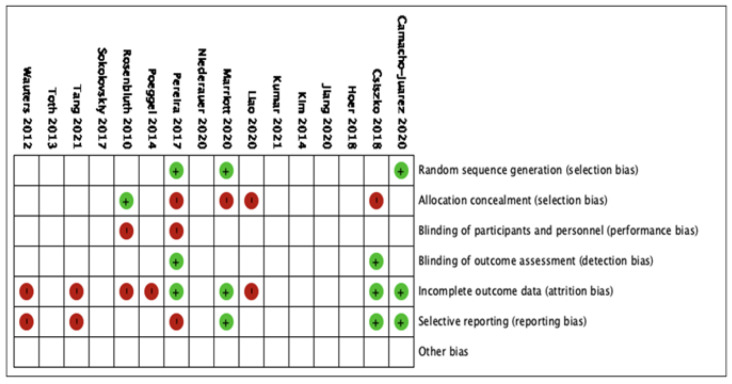
The risk of bias analysis of included studies.

The 16 studies were included in the qualitative analysis. No relevant applicability concerns were detected in any study. The κ coefficient for the agreement between reviewers was 0.78. The quality of the review was assessed according to QUADAS-2, and the risk of bias is summarized in Figure 3. The bias of individual studies is listed in Figure 4.

### 3.2. Study Characteristics

#### 3.2.1. Study Type and Developing Stage

**Figure 5:**
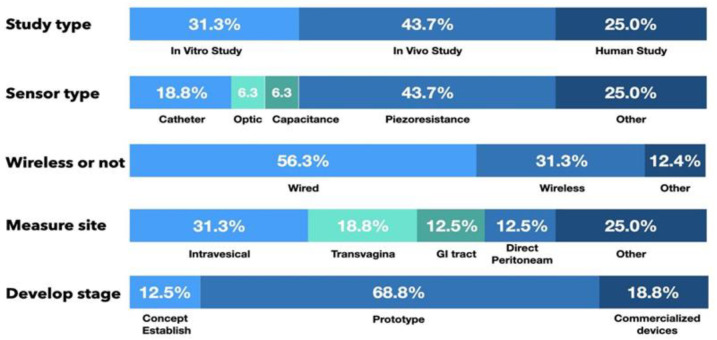
The characteristic of the included studies.

Of the 16 articles that report the measurement of IAP, there were 5 in vitro studies (31.3%), 7 animal studies (43.7%), and 4 human clinical trials (25.0%). In addition, 2 studies were in the proof-of-concept stage (12.5%), 11 studies presented a prototype sensor (68.8%) to measure IAP, and 3 studies used commercialized devices to detect IAP (18.8%), as shown in Figure 5.

#### 3.2.2. Sensing System and Measurement Route

Piezoresistive sensor systems were the most frequently reported sensor type, with seven studies using piezoresistance-based sensors (43.7%). Another three studies used air-/fluid-filled catheter-based sensors (18.8%), one study used a diaphragm displacement system (6.3%), one study focused on a microfluidic-based sensor (6.3%), and one study used an optic-based sensor to detect IAP (6.3%).

With the advancement of wireless communication technology, wireless sensing systems are increasingly being developed. In this review, nine studies used wired sensing systems (56.3%), and another five studies presented wireless sensing systems (31.3%) to monitor changes in IAP.

Among the IAP measurement routes for the reported sensors, two studies (12.5%) involved direct peritoneal cavity catheter insertion. Five studies (31.3%) used intravesical sensors, three (18.8%) used intravaginal sensors, one article described an intragastric sensor (6.3%), and another presented a capsular sensor in the gastrointestinal (GI) tract to detect IAP (6.3%). Another three studies used another route to detect changes in pressure or indirectly calculated IAP with images or skin resistance. From the available data, measurements taken by sensor systems in the vagina and GI tract had a high correlation with IAP and a high sensing resolution, as shown in Table 4.

## 4. Discussion

In this systematic review, we found numerous articles presenting novel designs of pressure sensors that detect IAP in a variety of ways. Changes in the measurement route and the increasing use of wireless signal delivery are the most notable findings of this review. IAP is a critical physiological parameter that changes regularly during various activities. Because the peritoneal cavity can be considered a closed space [2], intravesical pressure is currently the most frequently used route to detect IAP. However, changes to the volume of the cavity due to body position [56], muscle composition and contraction [57], or respiration can substantially change IVP [7], which limits the accuracy and precision of IVP devices. Kumar et al. [30] reported a stretchable capacitive pressure-sensing sleeve that can detect the pressure change on the Foley tip, which might further improve IVP measurement. Groups from the University of Utah [2,4,45,52] presented several studies and devices to measure IAP through the transvaginal route. These devices can continuously measure IAP without limiting the activity of participants. The GI tract is another route to detect IAP [17]. In contrast to the bladder and vagina, the GI tract is actually in the peritoneal cavity, which is more representative of the IAP. However, the correlation between intraluminal pressure and IAP is still debated. Liao et al. [33] presented an animal study demonstrating that the correlation between GI intraluminal pressure and IAP was better than that between IVP and IAP [33]. There are still other routes to detect IAP, such as surface resistance calculation [55] or image estimation [49,58]. Since there were no direct pressure measurements, we do not discuss the excellent results of these methods herein. The sensor presented by Jiang et al. is a pressure-sensitive membrane on a reservoir containing microfluid, and the in-channel fluid displacement is proportional to the applied pressure. However, ex vivo experimental results revealed a spatial resolution of 9 mmHg/30 µm. The pressure resolution is dependent on the resolution of the ultrasound transducer [54].

**Figure 6:**
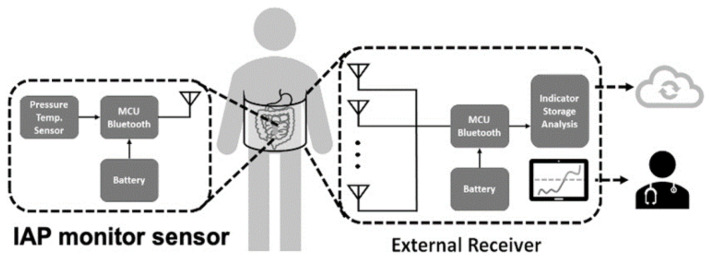
Schematic of wireless intra-abdominal pressure-monitoring sensor platform that can deliver information to clinical healthcare providers and cloud-based databases.

Wireless sensor monitoring devices are an innovative approach when developing physiological monitors. With the advance of antenna and communication technology, wireless signal transmission using Bluetooth or WiFi makes remote monitoring feasible [33,45]. One commonly available wireless system consists of an implanted sensor and external receiver, in which internal power provides high data bandwidth at ranges of 0.2–5 m. This wireless scheme requires internal batteries, which need to be recharged or replaced and oftentimes increase the implant size. Other wireless strategies utilize a passive telemetry approach for power and data transfer. These systems employ electromagnetic coupling and utilize backscatter amplitude modulation to detect signal changes [59]. Remote IAP monitoring is exemplified by the system presented by Liao et al. [33], which continuously measures pressure changes for up to 144 h, operating without restricting the patient’s activity and ambulation. Moreover, with this tool, signals can be collected in a central database on-site or even in the cloud, facilitating digital health management. Integrated wireless sensor systems depend on the special electrical circuitry, power supply, and antenna design and sometimes need specialized chips to effectively operate in the human body, which is a technological barrier to transitioning from conventional IAP monitoring devices, as presented in Figure 6.

**Figure 7:**
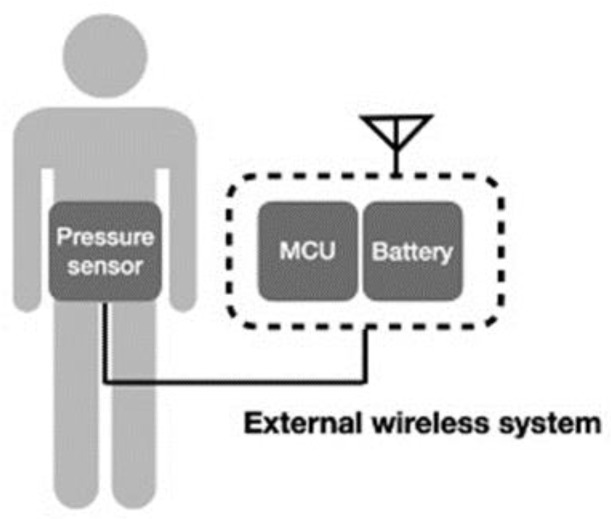
Schematic of external wireless intra-abdominal pressure-monitoring sensor system.

Another wireless system incorporates the conventional device into a wireless system [48,52], which can also deliver signals wirelessly, as illustrated in Figure 7. In this system design, the wireless component does not need to be minimized or composed with the sensor. Transducers based on MEMS for physiological pressure monitoring are used in conjunction with wireless telemetry techniques to transfer pressure data measured within the body to an externally located receiver. Telemetry systems are available from several manufacturers for both clinical and research use. Thus, the technological challenge can be reduced, and continuous data can be transmitted. However, users may have difficulty with the restriction of activity and discomfort.

Currently, IAP can be assessed by placing sensors directly into the abdominal compartment, gastrointestinal tract, bladder, and vagina [60]. The drawbacks of wired-based methods are their invasiveness or requirement to maintain a strict sensor orientation. Additionally, the current sensing equipment limits the natural movement of patients [61], and the IAP data are influenced by activity [4,7]. All of these limitations discourage physicians from measuring IAP routinely or earlier in the critical care unit. The potential advantages of wireless sensors include not restricting the ambulation of patients [45] and avoiding the risk of accidental dislodgement of wired sensors [62]. In this review, four wireless IAP sensors that can detect IAP from the GI tract, suture line, and vagina were identified.

Pressure sensor resolution has become a critical consideration in sensor design and selection. The abdominal compartment normally maintains a pressure of approximately 5–7 mmHg [63,64], and many pathological conditions can generate sustained pressures greater than 12 mmHg. Changes in IAP can cause a range of physiological and pathophysiological reactions. For example, elevated IAP decreases capillary blood flow in visceral organs and can lead to significant morbidity and mortality in critical care medicine. Therefore, 50 Pa (0.5 cm H_2_O) has been regarded as the acceptable resolution of intravesical pressure sensors [22,65]. For sensors intended for other routes, this parameter is not well defined. Our review revealed that the pressure resolutions of sensors were about 3.5 cm H_2_O for the transvaginal route and 0.1 cm H_2_O for the GI tract, as shown in Table 3. This sensing resolution was most commonly obtained using catheter-based and piezoresistive-type sensors. A catheter with a fluid-filled pressure transducer is the traditional method for checking intravesical pressure, and in this situation, the urine is the uncompressed fluid to measure the pressure gradient. However, precision pressure can be achieved with the piezo-type sensor, which can record pressure with a resolution of 0.1 cm H_2_O. Physiological pressure measurement systems typically employ electromechanical transducers that convert pressure into an electrical signal that can be processed. Historically, devices that were used to develop clinical standards for such measurements utilized micromachined piezoresistive sensors, which have high accuracy and tiny size.

There are also some passive methodologies to measure IAP. The advantages of passive sensors are long-term use, no battery, small size, and simple structure. However, the accuracy and resolution of passive sensors can only meet basic requirements and can be easily affected by the environment. The design of the external sensor reader is also a challenge because of the tissue effect.

Jiang et al. [54] presented a sensor with a pressure-sensitive membrane on a reservoir containing microfluid, and the in-channel fluid displacement is proportional to the applied pressure. The researchers measured IAP by using ultrasound to visualize the gray-scale image of fluid displacement for wireless and passive pressure monitoring. The ex vivo experimental results revealed a spatial resolution of 9 mmHg/30 µm. The pressure resolution is also dependent on the resolution of the ultrasound transducer.

Another popular passive pressure sensor is based on the resonance of an inductor (L) and a capacitor (C), and an external readout antenna coil can couple the LC resonator. The LC resonance frequency decreases as the applied pressure increases the capacitance of the sensor. Benken et al. developed a capsule that consists of an inductor coil wound around a cylinder and a capacitive pressure transducer. The resolution was up to 0.8 mmHg in vivo for a coupling distance of only 6 cm, which is highly related to the size and quality factor of the LC resonator [66].

The design of IAP-measuring sensors has been modified for different target patients. For open-abdomen or postoperative patients, the direct peritoneal sensor is relatively easy to insert, whether in adult or pediatric patients [67,68]. However, for patients without surgical intervention or those who are critical, the insertion of a direct peritoneal catheter will increase the possibility of peritoneal viscus injuries. On the other hand, for patients with a pelvic floor disorder, an intravaginal wireless device can continuously and comfortably measure IAP without the limitations imposed by wired and heavy sensing systems. It is difficult to apply these sensors to male patients to detect IAP. Therefore, the ability to use the device for all the patients is another consideration during the product research and design stage. A wireless digestive tract device might offer advantages because it will not be limited by body position or sex differences. However, occlusion and retention in the GI tract are another consideration for this type of device [69,70].

### Limitation

This study is a systematic review of IAP-measuring sensors developed in the last decade. All available articles were reviewed and collected for the evaluation of contemporary practice. However, the review has some limitations. First, we did not collect non-English-based literature, which might lead to the omission of some articles. Our reviewers evaluated available abstracts; therefore, if a non-English article had an English abstract, we had also registered their data. Second, the definition of IAP is variable, which might influence the final distribution of our data. Third, some manuscripts were published 10 years ago, and the technology and resolution of the sensors reported therein are disparate from the current status of these diagnostic tools. Therefore, selective bias cannot be avoided entirely. Fourth, the review included no randomized controlled trials and only one prospective observational study on this topic, which might affect the evidence strength, making it less decisive. In this review, we identified several innovative IAP-sensing systems, and several were still in the development stage (proof-of-concept and prototyping). Therefore, the risk of bias during the evaluation of the studies cannot be ignored. Furthermore, the risk of bias in most studies is unclear, which leads to another limitation of this review.

## 5. Conclusions

In the last several years, an increasing number of studies have reported the development and validation of IAP-sensing systems. A variety of sensors with wireless technology have improved the quality and efficiency of measurements. The selection of sensors should be based, not only on the technology, but also on the needs of target patients.

## Figures and Tables

**Table 1:** The grading system of intra-abdominal hypertension.

Grade I	IAP 12–15 mmHg
Grade II	IAP 16–20 mmHg
Grade III	IAP 21–25 mmHg
Grade IV	IAP > 25 mmHg.

**Table 2:** Comparison of current intra-abdominal pressure measurement sensors.

Category	Water-/Air-Filled Catheter	Diaphragm Capacitance	Diaphragm Piezoresistance	Optical Fiber
Accuracy	5 cm H_2_O (normal case) 10 cm H_2_O (positioning angle = 45°) [28,29]	0.15–25 cm H_2_O [37]	0.1 cm H_2_O [33]	0.1 cm H_2_O [36]
Sensor selection	Natural tract available	Free movement of fluid available	High accuracy necessary	Minimal sensor location
Source of errors	Length, diameter, and compliance of the catheter material [38]	Linearity error [37]	Time drifts of the sensing resistance [39]	Blockade of light source [40]
Selection of instrument site	Clear fluidic space	Space with free fluid	Place of maximum stress [41]	Possibility of fiber insertion
Advantage	No external power. Current standard.	Robust	Robust, small	Accurate
Disadvantage	Low accuracy; labor intensive; risk of infection; variation from bowel perforation and peritonitis	Expensive system	Power consumption	Expensive; Wired system

**Table 3:** The characteristics of included studies about novel intra-abdominal pressure sensor systems.

Authors	Year	Sensor Model	Study Type	Wire or Wireless	Sensor Route
Rosenbluth et al. [4]	2010	Capsular piezoresistive sensor with a delivery wire	Human	Wireless	Transvaginal
Wauters et al. [44]	2012	Intragastric tube tip	Animal study	Wired	Transgastric
Coleman et al. [45]	2012	Transvaginal piezoresistive sensor	Human	Wireless	Transvaginal
Tóth et al. [46]	2013	Piezoelectric sensor	In vitro	Wired	Non-applicable
Poeggel et al. [36]	2014	Fiber-Optic Pressure Sensors	In vivo	Wired	Transvesical
Kim et al. [47]	2014	Piezoelectric coil loop with a ferrite core	In vitro and in vivo	Wired	Transvesical
Sokolovskiy et al. [48]	2017	Wireless system connected to conventional urinary catheter	In vitro	Non-applicable	Transvesical
Pereira et al. [49]	2017	Ultrasonography to detect the IVC size	Human study	Other	Body surface
Csiszkó et al. [50]	2018	Direct pressure sensor to open-abdomen	Animal study	Wired	Direct peritoneal cavity
Höer et al. [51]	2018	Tension sensor on the suture	Animal study	Wireless	Suture line
Niederauer et al. [52]	2019	Transvaginal piezoresistive sensor attached to a speculum	In vitro	Wired	Transvaginal
Liao et al. [33]	2020	Wireless ingestible piezoelectric sensor	In vivo	Wireless	Gastrointestinal tract
Camacho-Juarez [53]	2020	Hermetic chamber and two valves to achieve pressure measurement	In vitro and human study	Wired	Transvesical
Jiang et al. [54]	2020	Microfluid-based displacement sensor	In vitro study	Wireless	Direct peritoneal cavity
Kumar et al. [30]	2021	Capacitive sensor fixed on the tip of Foley catheter	In vitro study	Wired	Transvesical
Tang et al. [55]	2021	Piezoresistive strain pressure transducer on skin	In vivo	Wired	Body surface

**Table 4:** Studies with intra-abdominal pressure-sensing systems with routes other than intravesical pressure measurement with detailed pressure resolution.

Author	Year	Route/Type	Study Type	Pressure Resolution	Comparison with Direct Intraperitoneal Sensing Calibration	Target Patients
Rosenbluth et al. [4]	2010	Vaginal/Wireless	Human	±3.5 cm H_2_O	High correlation	Female pelvic floor disorder
Coleman et al. [45]	2012	Vaginal/wireless	Human	±3.5 cm H_2_O	+ High correlation	Female pelvic floor disorder
Wauters et al. [44]	2012	GI tract/wired	Animal study	±2.6 cm H_2_O	Moderate correlation	Critical patients
Csiszkó et al. [50]	2018	Peritoneal cavity/Wired	Animal study	Not provided	High variation between different sensors	Postoperative open-abdomen patients
Niederauer et al. [52]	2020	Vaginal/Wireless	Human	±3.5 cm H_2_O	+ High correlation	Female pelvic floor disorder
Liao et al. [33]	2020	GI tract/Wireless	Animal study	±0.1 cm H_2_O	+ High correlation	Critical patients

## Data Availability

The data presented in this study are available on request from the corresponding author. The data are not publicly available due to the restriction of local law and government policy.
